# Delayed low-dose methotrexate excretion in a rheumatoid arthritis patient: A case report and literature review

**DOI:** 10.1097/MD.0000000000037070

**Published:** 2024-01-26

**Authors:** Qingzi Yan, Haibo Lei, Ting Gong, Renzhu Liu, Xiang Liu

**Affiliations:** a Department of Clinical Pharmacy, Xiangtan central hospital, Xiangtan, China.

**Keywords:** case report, delayed excretion, methotrexate, rheumatoid arthritis

## Abstract

**Rationale:**

Low-dose methotrexate has a relatively good safety profile. However, in cases where patients with multiple risk factors, a delayed excretion has been observed, resulting in the occurrence of severe adverse reactions. It is necessary to supervise and intervene throughout the entire process of treating patients with multiple risk factors for methotrexate, and to strengthen the rational application of methotrexate.

**Patient concerns and diagnoses:**

A 66-year-old male patient was admitted to our hospital with rheumatoid arthritis and underlying conditions such as chronic obstructive pulmonary disease (COPD). This patient received treatment with low-dose MTX (10 mg/week) and experienced adverse reactions including anemia. He was diagnosed with methotrexate-induced bone marrow suppression.

**Interventions and outcomes:**

The therapeutic drug monitoring revealed that the serum drug concentration of methotrexate was at a critical level and the patient was rescue with calcium folinate and other adjuvant therapy such as transfusions of red blood cells, plasma, platelets, oral Yixuesheng tablets and Leucogen tablets. We conducted a 1-month follow-up, and there was no recurrence of bone marrow suppression and anemia.

**Lessons:**

To ensure rational administration of methotrexate, it is important to fully evaluate the clinical manifestations and physical condition of patients and regularly detecting the serum drug concentration of methotrexate when patients with multiple risk factors, Otherwise, even low-dose methotrexate administration may cause delayed excretion, resulting in severe adverse reactions.

## 1. Introduction

Rheumatoid arthritis (RA) is a disease defined as a chronic and systemic autoimmune disease involved in joints and surrounding tissue damage associated with a chronic inflammatory process.^[1]^ Additionally, it may cause extra-articular manifestations, such as subcutaneous nodules, anemia, heart, kidney, lung, digestive system, eye, skin, nervous system, pulmonary interstitial lesions and vasculitis.^[2]^ RA may even lead to apparent joint damage and progresses to limb disability and dysfunction without system therapy. It is exerted a negative impact on the life quality of patients and produces a substantial social burden. Methotrexate (MTX) is the recommended first-line conventional anti-rheumatic drug for treating RA. It is an artificial compound and folic acid analogue that competitively inhibits dihydrofolate (FH2) and dihydrofolate reductase (DHFR) to reduce intracellular folic acid (FH4) levels. This ultimately affects the metabolism of purine and pyrimidine^.[3]^ It is administered mainly through oral route, intramuscular injection, and subcutaneous injection. The oral MTX absorption range is quite wide when used to treat RA in different individual. Generally speaking, MTX absorption is relatively superior at low doses, with good safety and tolerance.^[2]^ Delayed low-dose MTX excretion is a rare entity in the literature, and this article reports a case of bone marrow suppression caused by delayed excretion of low-dose MTX, warning to medical staff and patients should strengthen the rational application of MTX.

## 2. Case presentation

A 66-year-old male patient was admitted to our hospital with recurrent cough, expectoration for 5 years, breathlessness for more than 1 year, and aggravation for 5 days, he was referral from another hospital to us with no systematic treatment. There are various kinds underlying diseases with this patient, including chronic obstructive pulmonary disease, pulmonary fungal infection, rheumatoid arthritis, and double kidney stones. Moreover, this patient received low dose MTX (10 mg/w) treatment for RA, the last dose was administered before last week.

Physical examination indicated the patient body temperature was 36.8°C, blood pressure was 100/65 mm Hg, and heart rate was 77/min. X-ray examination showed that the patient had hydrothorax in the thoracic region and ascites in the abdominal area. Additionally, electrolyte abnormalities, abnormal renal function, pancytopenia, and medium anemia were reported based on laboratory examination that the patient red blood cell count (RBC) was 2.03 × 10^12^/L, blood platelet count (PLT) was 20.0 × 10^9^/L, white blood cell count (WBC) was 1.80 × 10^9^/L, hemoglobin (HGB) level was 62.0g/L, platelet distribution width (PDW) was 17.80fl, hematocrit (HCT) was 18.3%, monocyte percentage (MO%) was 2.7%, and red blood cell distribution width-coefficient of variation (RDW-CV) was 15.20% respectively. The patient exhibited a significant reduction in blood cell count such as RBC, WBC, PLT, HGB, and HCT, indicating the presence of anemia. This condition could be attributed to bone marrow suppression resulting from delayed excretion of MTX. Furthermore, the patient developed gastrointestinal symptoms such as diarrhea and obstruction due to severe intestinal inflammation, infection, and infectious exudative peritoneal effusion. No thoracalgia, no hematochezia, no drug abuse and other symptoms were reported.

**Figure 1.**
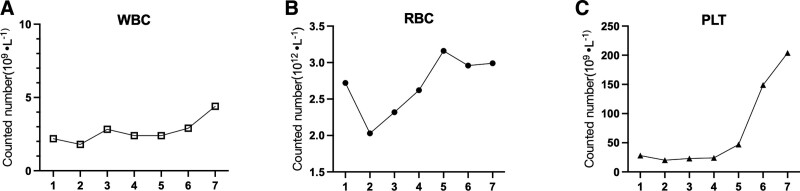
The variations of hemocyte throughout patient hospitalization period.

After admission, the patient received transfusions of red blood cells, plasma and platelets. At the same time, oral Yixuesheng tablets^[4]^ were administered to improve anemia, and Leucogen tablets were used to regulate white blood cell levels. Antibiotics such as meropenem and cephalothin were administered to control the infection. Proton pump inhibitors (PPIs) such as omeprazole, rabeprazole or pantoprazole were used to treat stress ulcer. Dexketoprofen was used to relieve the joint pain in the patients’ hands. Additionally, the serum concentration of MTX was 4.07 µmol/L detected by 2-dimensional liquid chromatography (HPLC, LC-20AT, Shimadzu, Japan); (lC2801 coupling instrument, Demeter, China). It is speculated that the patient experienced bone marrow suppression and pancytopenia due to delayed excretion of MTX. Therefore, MTX was discontinued and calcium folinate rescue was initiated. The patient weighed 47.5 kg and was 156 cm tall, resulting in a calculated body surface area of approximately 1.44 m^2^. The patient was administered 25 mg of folic acid every 6 hours, and the urine was alkalized with sodium bicarbonate and properly hydrated. Three days later, the blood concentration of MTX was detected again and found to be 0 μmol/L or lower than the detection limit. As shown in Figure 1, WBC, and PLT of patient are returned to normal range on the sixth day after rescue with calcium folinate, and RBC recovered to normal during the subsequent treatment. The other symptoms including cough, shortness of breath, infection improved after 20 days of systematic treatment. The patient was subsequently discharged and prescribed medications to treat the underlying conditions. Additionally, regular follow-up appointments were scheduled.

## 3. Discussion

### 3.1. Correlation evaluation of MTX adverse reactions

Following the administration of MTX, this patient experienced adverse reactions such as bone marrow suppression and anemia. According to the “Adverse Drug Reaction Terminology Guide” provided by the National Adverse Drug Reaction Monitoring Center, the evaluation of adverse drug reactions encompasses 5 criteria. Firstly, the temporal correlation between medication and reaction was deemed rational, as the patient manifested bone marrow suppression and anemia 4 weeks after MTX utilization. Furthermore, the observed reaction was consistent with established adverse reactions associated with MTX administration, namely bone marrow suppression and anemia. These reactions have been frequently reported in patients receiving MTX administration. Following the cessation of MTX, the reaction gradually diminished and symptom improvement, as well as eventual disappearance of the patient symptoms after the implementation of calcium folinate therapy. It is important to acknowledge that the utilization of antibacterial medications, such as meropenem and cephalothin, may also contribute to the development of pancytopenia. Therefore, the correlation evaluation aligns with the 3 items of 5 criteria, making it highly correlated to anemia, bone marrow suppression and the adverse reactions of MTX in this patient.

### 3.2. Risk factors of delayed MTX excretion

According to the guidelines for RA treatment, stated by both EULAR (European League Against Rheumatism) and ACR (American College of Rheumatology), it is strongly advised to begin treatment promptly with traditional anti-rheumatic drugs (csDMARDs), MTX is recommended as the initial medication of choice.^[2,5,6]^ Studies have shown that only 35%-50% of patients treated with MTX exhibited a good clinical response, and about 30% had to discontinue the treatment due to adverse reactions.^[7]^ Methotrexate (MTX) is characterized by a range of adverse reactions, including gastrointestinal, liver, and kidney damage. Prolonged use of this medication can also result in cough, shortness of breath, and interstitial pneumonia. Among these reactions, bone marrow suppression is considered a significant and serious side effect.^[8]^

The half-life of MTX typically ranges from 2 to 3 hours, with a peak blood concentration observed within 1 to 2 hours following oral administration. It is primarily excreted in its original form from renal, with <10% excreted through bile. However, certain active metabolites such as MTX, 7-Hydroxy-methotrexate (7-OH-MTX), and MTX-polyglutamate (MTX-PG) may be stored in organs such as the kidneys and liver for weeks to months, resulting in significant individual variation in clearance rates.^[9]^ Delayed excretion of MTX is more commonly observed in high-dose usage, however, low-dose MTX usage could provoke delayed excretion particularly in the influence of multiple risk factors, leading to adverse reactions such as nephrotoxicity, hematotoxicity, hepatotoxicity, and mucosal damage. In the case of this patient, low-dose MTX was taken for RA, caused adverse reactions including bone marrow suppression and anemia, the main clinical symptom is the blood cell count lower than normal reference value. The patient with many underlying diseases, such as bilateral kidney stones, renal dysfunction, pleural and peritoneal effusion, etc. It may lead to MTX accumulation and delayed excretion, which further aggravates renal function damage and forms a vicious circle, resulting in clearance time significantly extended. In addition, the presence of pleural effusion and ascites in the patient significantly reduces the clearance rate of MTX, extending its half-life and increasing the risk of delayed excretion.^[10]^ This would be another reason for delayed MTX excretion in this patient. Moreover, the metabolic process of MTX is complex and susceptible to other combined drugs. This patient also has a history of using PPIs (such as omeprazole, pantoprazole, and rabeprazole), NSAIDs (ibuprofen, dexketoprofen, etc), and antibiotics (meropenem, cefalotin, etc) during MTX treatment and hospitalization. MTX and its metabolites are eliminated through the renal tubules by hydrogen-potassium-ATPase hydrogen ions. However, PPIs inhibit the elimination process of hydrogen ions in the kidneys, potentially inhibiting the excretion of MTX and 7-hydroxymethotrexate.^[11]^ Similarly, NSAIDs may reduce the secretion of MTX and its metabolites in renal tubules by competitively binding with anion transporters, thereby delaying their excretion.^[12]^ Furthermore, the use of antibiotics, such as meropenem and cefalotin, during the patient hospitalization has also contributed to the delay in the excretion of MTX.^[13]^ Consequently, even after discontinuing MTX, the patient still experienced symptoms of anemia, suggesting a high concentration of residual drugs. Therefore, it is important to regularly monitor the blood concentration of MTX and initiate appropriate measures based on the results. For this paient, we detected the serum drug concentration was crisis value and initiated the rescue immediately. The patient symptoms of anemia were effectively alleviated through the administration of calcium folinate rescue and symptomatic treatment. Additionally, genetic polymorphisms, such as 5,10-methylenetetrahydrofolate reductase (MTHFR), reduced folate carrier (RFC1), glutathione S-transferase (GSTP1), ATP-binding cassette transporter (ABC), gamma-glutamyl hydrolase (GGH), and follylpolyglutamate synthase (FPGS), have been found to significantly influence MTX metabolism and excretion.^[14,15]^ However, there is controversy regarding the association between gene polymorphism and the delay in MTX excretion, more studies are needed to explore the effect of gene polymorphism on MTX. Unfortunately, the patients in this case did not carry out the detection of related genes, and it was impossible to judge the correlation between low-dose MTX delayed excretion and genes in this patient.

## 4. Conclusion

Before initiating MTX treatment, doctors should comprehensively assess the patient clinical manifestations and physical condition. Patients receiving MTX therapy require regular follow-up, including blood cell count, liver and kidney function tests. Therapeutic drug monitoring can be used as a diagnostic tool for delayed MTX excretion in patients with multiple risk factors. Medication adjustments should be made according to serum drug concentration. Otherwise, even with low doses of MTX, delayed excretion may still occur, leading to adverse reactions. Patients with suspected adverse reactions to MTX should undergo timely detection of serum drug concentration to determine whether there is delayed excretion of MTX. Of course, it is necessary to acknowledge the limitations of this work. Because this study aimed to investigate a rare adverse reaction of low-dose MTX in a specific population, we had access to a limited sample size, which may have affected the generalizability of this work, additional prospective studies are needed to substantiate our claim.

## Author contributions

**Conceptualization:** Qingzi Yan.

**Funding acquisition:** Ting Gong.

**Investigation:** Qingzi Yan, Ting Gong.

**Methodology:** Qingzi Yan.

**Supervision:** Xiang Liu.

**Visualization:** Haibo Lei.

**Writing – original draft:** Qingzi Yan.

**Writing – review & editing:** Renzhu Liu.
